# In vitro and in vivo antibiofilm activity of the synthetic antimicrobial peptide WLBU2 against multiple drug resistant *Pseudomonas aeruginosa* strains

**DOI:** 10.1186/s12866-023-02886-x

**Published:** 2023-05-15

**Authors:** Sara Masihzadeh, Mansour Amin, Zahra Farshadzadeh

**Affiliations:** 1 Infectious and Tropical Diseases Research Center, Health Research Institute, Ahvaz Jundishapur University of Medical Sciences, Ahvaz, Iran; 2 Department of Microbiology, School of Medicine, Ahvaz Jundishapur University of Medical Sciences, Ahvaz, Iran

**Keywords:** Antimicrobial peptides, WLBU2, Biofilm, *Pseudomonas aeruginosa*

## Abstract

**Background:**

The global crisis of antibiotic resistance increases the demand for the novel promising alternative drugs such as antimicrobial peptides (AMPs). Here, the antibiofilm activity of the WLBU2 peptide against *Pseudomonas aeruginosa* (*P. aeruginosa*) isolates was investigated in this study.

**Methods:**

Two clinical MDR and carbapenem resistant *P. aeruginosa* (CRPA) isolates, and standard *P. aeruginosa* ATCC 27,853 were investigated. The MIC and MBC of WLBU2 were determined. The MBIC was determined to evaluate inhibitory activity of WLBU2 on biofilm formation and MBEC to dispersal activity on preformed biofilm. The relative expression levels of biofilm-associated genes including *rhlI*, *rhlR*, *lasI* and *lasR* were analyzed using RT-qPCR. In vivo evaluation of inhibitory effect of WLBU2 on biofilm formation was performed in the murine models of *P. aeruginosa* biofilm-associated subcutaneous catheter infection.

**Results:**

MIC and MBC of WLBU2 for both MDR and ATCC 27,853 *P. aeruginosa* strains were 8 and 16 µg/mL, respectively, while both the MIC and MBC against the CR strain were 4 µg/mL. MBIC was estimated to be 64 µg/ml for all strains. MBEC against MDR and ATCC 27,853- *P. aeruginosa* strains was 128 µg/ml and against CRPA was 64 µg/ml. The bacterial adhesion to a static abiotic solid surface (the surface in the polypropylene microtiter wells) was significantly inhibited at 1/4× MIC in all *P. aeruginosa* strains and at 1/8× MIC in CRPA strain (P < 0.05). Following treatment with WLBU2 at 1/8× MIC, significant inhibition in biofilm formation was observed in all isolates (P < 0.05). Results of the colorimetric assay showed that WLBU2 at 4× MIC was able to disperse 69.7% and 81.3% of pre-formed biofilms on abiotic surface produced by MDR and standard (ATCC 27,853) *P. aeruginosa*, respectively (*P* < 0.03), while a 92.2% reduction in the CRPA biofilm was observed after treatment with 4× MIC WLBU2 (P < 0.03). The expression levels of all genes in isolates treated with 1/2 MIC of WLBU2 were down-regulated by more than four-fold compared to the untreated isolates (*P* < 0.05). WLBU2 significantly inhibited biofilm formation in murine catheter-associated CRPA infection model at 1/4×MIC, 1/2×MIC, and 1×MIC by 33%, 52%, and 67%, respectively.

**Conclusion:**

Considering relatively strong inhibitory and eradication potency of WLBU2 on the *P. aeruginosa* biofilms in in vitro and in vivo conditions, the peptide can be considered as a promising candidate for designing an antibiofilm drug.

## Introduction

Multidrug-resistant (MDR) bacteria are a serious threat to public health, especially in healthcare settings [1, 2]. It has been estimated that MDR infections kill 700,000 people globally every year [3]. Among MDR bacteria, *Pseudomonas aeruginosa* (*P. aeruginosa*) is the most frequent causative agent in healthcare associated infections. The World Health Organization (WHO) has listed this bacterium as one of the greatest threats to human health [4]. *P. aeruginosa*, a Gram-negative opportunistic pathogen, is responsible for serious infections causing substantial morbidity and mortality. The bacterium causes several community- and hospital-acquired life-threatening infections such as pneumonia, bloodstream, meningitis, and wound infections [5, 6].

Bacterial biofilms consist of multilayered, organized, localized and heterogenous communities of bacteria surrounded by a self-produced matrix of extracellular polymeric substances (EPSs) [7, 8]. EPSs are composed of biomolecules such as polysaccharides, proteins, extracellular DNA, and lipids [9]. Based on the reports published by the National Institutes of Health in the USA, biofilms are associated with 80% of human bacterial chronic infections [10]. Compared with planktonic mode, biofilm mode is more tolerant to the current standards of care and is typically associated with chronic infections, indicating that biofilm-forming ability is one of the most clinically important virulence determinants.

Biofilm formation can enhance the antimicrobial resistance of microorganisms by up to 500–50,000 times [9]. In general, *P. aeruginosa* isolates have intrinsic and acquired resistance to several classes of antibiotics such as fluoroquinolones, carbapenems, aminoglycosides, and β-lactams [11]. *P. aeruginosa* clinical isolates that produce biofilms are very difficult to treat in infections. Therefore, to overcome the antibiotic resistance crisis, there is an urgent demand for the discovery and development of the new promising alternative drugs with a potent impact on biofilm-forming bacteria [12].

Natural antimicrobial peptides (AMPs) are small, cationic peptides produced by different multicellular organisms, such as microbes, mammals, insects, and plants [13].AMPs have a wide range of antimicrobial activity and play a significant role in the nonspecific innate defense system against invading microbes. These components are attractive alternatives to classical antibiotics for the treatment of drug-resistant bacterial infections [14].Moreover, AMPs have strong activity against MDR bacterial biofilms [15]. AMPs have several specific features including disruption of the microbial cytoplasmic membrane, quick killing effect, little host toxicity, and low potential to induce and develop resistance [10]. AMPs can exhibit antibiofim activity through inhibition of bacterial adhesion to surfaces and reduction expression of various genes associated with quorum sensing, matrix synthesis or motility [16].

A cationic synthetic peptide, WLBU2, has been shown to has good antibacterial activity against Gram-positive and Gram-negative bacteria [17]. However, antibiofilm activity of the WLBU2 peptide on *P. aeruginosa* has not been fully investigated up to now. Here, we aimed to investigate the antibiofilm effects of the WLBU2 peptide against two drug-resistant *P. aeruginosa* isolates.

## Materials and methods

### Ethics statement

The present research was approved by the ethics committee of Ahvaz Jundishapur University of Medical Sciences (Ethical code: IR.AJUMS.ABHC.REC.1399.015). All experiments in this study were performed in accordance with ARRIVE guidelines (https://arriveguidelines.org).

### Synthesis and preparation of the WLBU2 peptide

Antimicrobial peptide WLBU2 (RRWVRRVRRVWRRVVRVVRRWVRR) (purity ≥ 95%) was purchased from Gil Biochemical Co., Ltd., Shanghai, China. WLBU2 stock solutions (1 mM) were prepared in sterile Milli-Q water and stored at − 20 °C until usage. The 2-fold serial dilutions of WLBU2 in the broth microdilution methods were prepared in Mueller–Hinton broth (MHB).

### Bacterial strains

In the current study one *P. aeruginosa* standard strain (ATCC 27,853) obtained from Iranian Biological Resource Center, Tehran, Iran and two *P. aeruginosa* clinical strains (one carbapenem resistant and one MDR strain) were obtained from the our previous study in which final identification of strains was performed using PCR of the gyrB gene [18]. All strains were obtained from stock cultures preserved at -80 °C in Tryptic Soy Broth (TSB) (Oxoid, Basingstoke, UK) containing 20% glycerol.

### The minimum inhibitory concentration (MIC) and minimum bactericidal concentration (MBC)

The broth microdilution method was used to determine MIC of the WLBU2 peptide against *P. aeruginosa* according to CLSI guidelines [19]. Overnight *P. aeruginosa* cultures were diluted in fresh Luria Bertani (LB) broth to give a final density of 5 × 10^5^ CFU/ml and added to wells of a 96-well polypropylene microtiter plate, containing two-fold WLBU2-dilutions ranging from 0.39 to 100 µM (0.39, 0.78, 1.56, 3.13, 6.25, 12.5, 25, 50, 100 µM) and incubated for 24 h at 37 °C. The MIC was considered as the lowest concentration of peptide at which no visible growth is seen, while MBC of WLBU2 as the lowest concentration of antimicrobial that caused at least 99.9% killing of the initial inoculums. MBC was determined by removing sample from every well at which no growth was observed for colony count assay on Mueller Hinton (MH) Agar plates [19].

### Time-kill assay

In the present study, time-kill assays were performed to investigate the killing rate of WLBU2 in comparison with colistin as a last-hope treatment for MDR gram-negative pathogens. For each strain, a final concentration of 10^7^ CFU/mL was prepared from overnight cultures and added to the 96-well microtiter plates (SPL life Sciences, Gyeonggi-do, Korea) in the presence of colistin (Sigma, USA) and WLBU2 at 1 *×* and 2 *×* MICs. The bacterial suspension of each strain without colistin and WLBU2 was considered as negative control. Following incubation at 37 °C for 0, 5, 10, 15, 20, 25 and 30 min, the colonies were counted to determine the number of CFU. Each sample was serially diluted and added to Mueller-Hinton (MH) agar (Merck, Germany). The lower limit of detection for the colony counts was 2 log10 CFU/ml. All tests were performed with three technical replicates of two biological replicates.

### Minimum biofilm inhibitory concentration (MBIC) and minimum biofilm eradication concentration (MBEC)

The effectiveness of WLBU2 was investigated based on its MBIC and MBEC. The MBIC represents the minimum concentration of the peptide that will prevent biofilm formation, while MBEC represents the minimum concentration of the peptide that can eradicate bacteria in biofilm mode. Inhibitory and eradicative activity of WLBU2 on biofilm were investigated in the presence of various concentrations of WLBU2 ranging from 0.39 to 100 µM (0.39, 0.78, 1.56, 3.13, 6.25, 12.5, 25, 50, 100 µM) using a static abiotic solid surface assay as previously described [20], with minor adjustment. Briefly, a 200-µl aliquot of 1:100 dilutions prepared from overnight LB culture of *P. aeruginosa* was added to each well in the presence of different concentrations of WLBU2 and incubated at 37 °C for 2 h (adhesion assay) or 24 h (MBIC) without shaking. LB culture of *P. aeruginosa* without the peptide was considered positive control. Following incubation, the plates were washed three times with 0.85% NaCl medium, and each well was stained with 200 µL of 0.1% crystal violet (CV) for 20 min at ambient temperature. The plates were again washed three times to remove excess dye, and incubated with 200 µl of 95% ethanol for 20 min. The absorbance of the crystal violet was then measured at 595 nm (OD_595_). Experiments were done in triplicate wells for each concentration and repeated three times.

In order to determine MBEC of WLBU2, the bacteria were cultured on a 96-well plate in the absence of the peptide. Following overnight incubation at 37 ^0^ C, 100 µl of each concentration of WLBU2 were added to each well. After overnight incubation, plates were washed with the 200 µl physiological saline solution. Then, 100 µl physiological saline solution was added to wells and biofilms formed by *P. aeruginosa* was scratched using a scalpel. Finally, 10 µl of the solution was taken from each well and plated on the MHA medium. All plates were incubated at 37 ^0^ C overnight. The lowest concentration at which we do not observe the growth of bacteria was considered MBEC.

### In vitro evaluation of dispersal activity of WLBU2 on biofilms

To investigate dispersal activity of WLBU2 on pre-formed biofilms, initially biofilms were allowed to develop through incubation of *P. aeruginosa* strains at 37 °C for 24 h in LB medium in a 96-well polypropylene microtiter plate. After biofilm formation, WLBU2 was added at 2 ×, 4 ×, and 8 × MIC and incubated at 37 °C for 12 h. Then, all non-adherent bacteria were removed through discarding the culture medium and rinsing the microtiter plate three times by PBS. After staining adherent biofilm biomass with 0.1% CV, the absorbance of the CV was measured at 595 nm using a microtiter plate reader [21]. Following crystal violet staining, measurement of absorbance of pre-formed biofilm in each strain and comparison with control, the percentages of dispersion of pre-formed biofilm were calculated.

### RNA extraction and complementary DNA synthesis

Total RNA was extracted from exponentially grown *P. aeruginosa* using the high pure RNA isolation kit (Roche Life Science) according to the manufacturer’s instruction. The quality and quantity of extracted RNA was evaluated using a NanoDrop spectrophotometer (ND-2000, Thermo Scientific, Loughborough, United Kingdom). The complementary DNA (cDNA) synthesis was carried out using the cDNA synthesis kit (Takara Bio, Japan).

### Semi quantitative RT-PCR

The relative expression levels of *rhlI*, *rhlR*, *lasI* and *lasR* genes were evaluated by RT-qPCR. The reaction was performed using the SYBR Select Master Mix (Ampliqon, Denmark) on the ABI Step One Plus machine (Applied Biosystem, USA). The primer sequences applied for the RT-qPCR reaction are listed in Table 1. RT-qPCR was performed in a total volume of 20 µl reaction including 10 µl of SYBR Select Master Mix, 1 µl of each primer (10 mM), 2 µl (∼1.2 µg) of template cDNA, and 6 µl of sterile distilled water. The relative expression of the genes was normalized against the *rpoD* gene. Results were calculated on the basis of the 2^−ΔΔCt^ method.

**Table 1:** Primer sequences used in the present study

Gene	Sequence		Product size (bp)
*lasI*	F	CGTGCTCAAGTGTTCAAGG	295
	R	TACAGTCGGAAAAGCCCAG	
*lasR*	F	AAGTGGAAAATTGGAGTGGAG	130
	R	GTAGTTGCCGACGACGATGAAG	
*rhlI*	F	TTCATCCTCCTTTAGTCTTCCC	155
	R	TTCCAGCGATTCAGAGAGC	
*rhlR*	F	TGCATTTTATCGATCAGGGC	133
	R	CACTTCCTTTTCCAGGACG	
*rpoD*	F	CATCCGCATGATCAACGACA	371
	R	GATCGATATAGCCGCTGAGG	

### Hemolysis assay

To perform hemolysis assay, the fresh human blood samples were received from returned unused blood bags in the blood bank (Iranian Blood Transfusion Organization) and used in accordance with the ethical standards. Following washing human red blood cells (RBCs) three times with PBS, cells suspension was prepared and incubated for 1 h at 37 °C in presence of WLBU2 at different concentrations (5, 25, 50, 100, and 200 µg/mL). After centrifugation of suspension and collection of the supernatant, the free hemoglobin in the supernatant was analyzed by UV–Vis spectrophotometer at 540 nm. The RBCs incubated with 0.1% Triton X-100 were used as 100% hemolysis (control).

### In vivo evaluation of inhibitory effect of WLBU2 on biofilm formation

The in vivo animal study was carried out based on the protocols and guidelines approved by the Institutional Animal Care and Use Committee (IACUC) of Sunchon National University (SCNUIACUC-2019-10).

Female BALB/c mice (6–8 weeks old and 18–22 g) were used to evaluation of inhibitory effect of WLBU2 on biofilm formation. The evaluation of inhibitory effect of peptide on biofilm formation was performed in different concentration including 1/8×MIC, 1/4×MIC, 1/2×MIC, and 1×MIC MIC. According to the G power formula, we divided mice to five group (five per group) and all mice were kept in ventilated cages at 22–25 ^0^ C. All mice were anesthetized by the intraperitoneal injection of ketamine (80 mg/kg) and xylazine (10 mg/kg). In order to place two polyurethane catheters, a small wound was created on the skin and catheters subcutaneously were inserted.

At the next step, we prepared the different concentrations (1× MIC, 1/2 MIC, and 1/4 MIC) of WLBU2 peptide. For this purpose, 1 µg/250µl, 2 µg/250µl, and 4 µg/250µl of peptide were mixed with 250 µl of CRPA suspension (10^6^ CFU/ml). The suspension was injected subcutaneously into groups 1 to 3 mice (each concentration in one group). The fourth group of mice was used as negative control and PBS was injected subcutaneously into this group. Moreover, the fifth group of mice was considered as positive control and bacterial suspension without WLBU2 peptide was injected subcutaneously into this group. Wounds were regularly disinfected with *povidone*-*iodine*. After 7 days, all mice were euthanized by the injection of ketamine and all catheters were removed from mice. In each mouse, biofilm formed on one catheter was stained with 250 µl of 0.5% crystal violet solution for 10 min and its absorbance (595 nm) was measured. To calculate percentages of biofilm dispersion in different peptide concentrations, the mean of absorbance (595 nm) in members of one group was compared with the mean of absorbance in control. Another catheter was investigated using a scanning electron microscope.

### Data analysis

All experiments were carried out in triplicate and data are expressed as the mean ± standard deviation. Comparisons between groups were statistically analyzed using variance (ANOVA) on the log-transformed data with Tukey–Kramer Hones Significant Difference Test. A *P* value less than 0.05 was considered as significant.

## Results

### Susceptibility of planktonic and biofilm- grown *P. aeruginosa* strains against WLBU2

To determine the antimicrobial activity of WLBU2 against *P. aeruginosa* strains the broth microdilution method was used according to the CLSI guideline. According to the results, the MIC and MBC values of WLBU2 against MDR and ATCC 27,853 *P. aeruginosa* strains were 8 and 16 µg/mL, respectively, while both MIC and MBC against carbapenem resistant strain was 4 µg/mL. The MBEC values were estimated to be 128 µg/ml against both MDR and ATCC 27,853 *P. aeruginosa* strains and 64 µg/ml against carbapenem resistant strain.

WLBU2 had a strong inhibitory and eradication effect on the *P. aeruginosa* biofilm; the antimicrobial peptide WLBU2 inhibited the biofilm of the strains with the strongest biofilm formation ability at 64–128 mg/ml, and can eradicate the biofilm at 256–512 mg/ml. MBEC results indicated that in order to completely remove the bacteria enclosed in the biofilm, a concentration several times higher than the MIC value of the peptide (2 to 4 fold higher than the MIC value for tested strains) was required.

### Time-dependent killing of *P. aeruginosa* by WLBU2

**Fig. 1:**
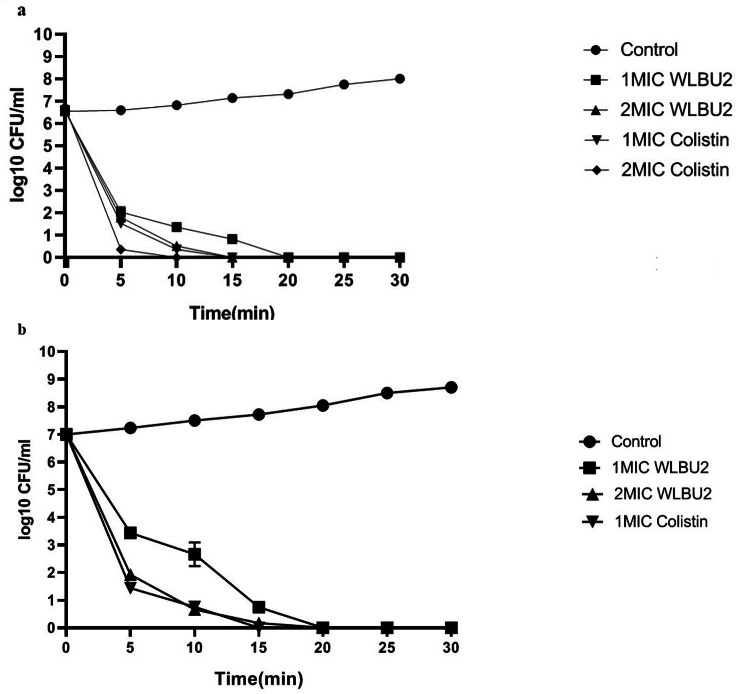
Time-Kill Kinetics of WLBU2 and Colistin against *P. aeruginosa.* 1 A: concentration-dependent killing of *P. aeruginosa* ATCC27853 by WLBU2 and colistin; 1B: concentration-dependent killing of CRPA by WLBU2 and colistin. There was no significant difference in the killing activity between WLBU2 and colistin (P > 0.05). All data represent mean ± standard error of the mean (SEM) of 3 independent experiments

The results of time-kill assays of WLBU2 and colistin against clinical isolates of *P. aeruginosa* (Fig. 1a, b) were identical to those against the standard strain, ATCC 27,853. The results of time-killing assay at 1 *×* MIC (4 *µ*g/mL) of peptide exhibited approximately a 6-log reduction in the viable count; regrowth was not observed after 24 h. At 1 *×* MIC (0.25 *µ*g/mL) of colistin, a 7-log reduction in the bacterial inoculum was obtained within 15 min. At 4 *×* MIC, the bactericidal activity of both agents was fast. WLBU2 and colistin at this concentration exhibited a 7-log reduction within 20 and 15 min, respectively. These results revealed that the killing activities of WLBU2 and colistin exhibited no significant difference at MIC and higher concentrations (P > 0.05).

### In vitro anti-adherence and anti‑biofilm potencies of WLBU2

Anti-adherence and anti-biofilm ability of WLBU2 against *P. aeruginosa* were determined using colorimetric assay.

**Fig. 2:**
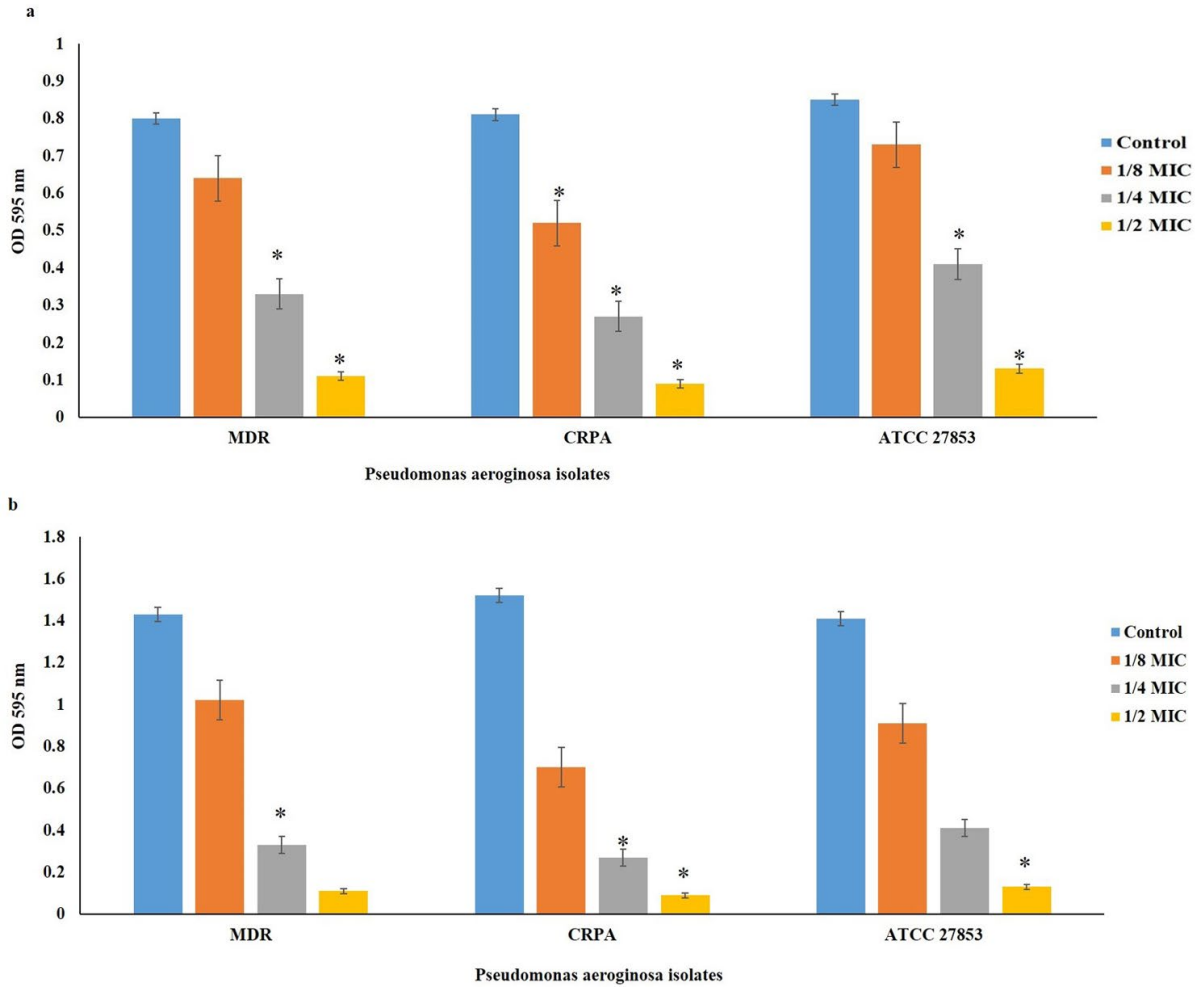
The effect of WLBU2 in different concentrations on bacterial attachment inhibition (**2a**) and biofilm formation inhibition (**2b**). Control is untreated strain and represent 100% biofilm formation. Data are mean ± SD. Statistical analyses were carried out by one-way analysis of variance (ANOVA) and Dunnett’s multiple comparisons test. Experiments with P < 0.05 were considered significant. P < 0.05 (∗), P < 0.01 (∗∗), P < 0.001 (∗∗∗), P < 0.0001 (∗∗∗∗), P > 0.05 non-significant compared to untreated positive controls

As presented in Fig. 2a, the bacterial adhesion was significantly inhibited at 1/2× and 1/4× MIC in all *P. aeruginosa* isolates compared with the control group (P < 0.05), whereas the inhibitory effect of WLBU2 at 1/8× MIC on bacterial adhesion was only observed for CRPA isolate (P < 0.05). On the other hand, following treatment with WLBU2 at 1/2×, 1/4×, and 1/8× MIC, significant inhibition of biofilm formation was observed in all isolates in comparison to the control (Fig. 2b; P < 0.05). MBIC was estimated to be 64 µg/ml for all strains. MBEC against MDR and ATCC 27,853- P. *aeruginosa* strains was 128 µg/ml and against CRPA was 64 µg/ml.

### Dispersal activity of WLBU2

**Fig. 3:**
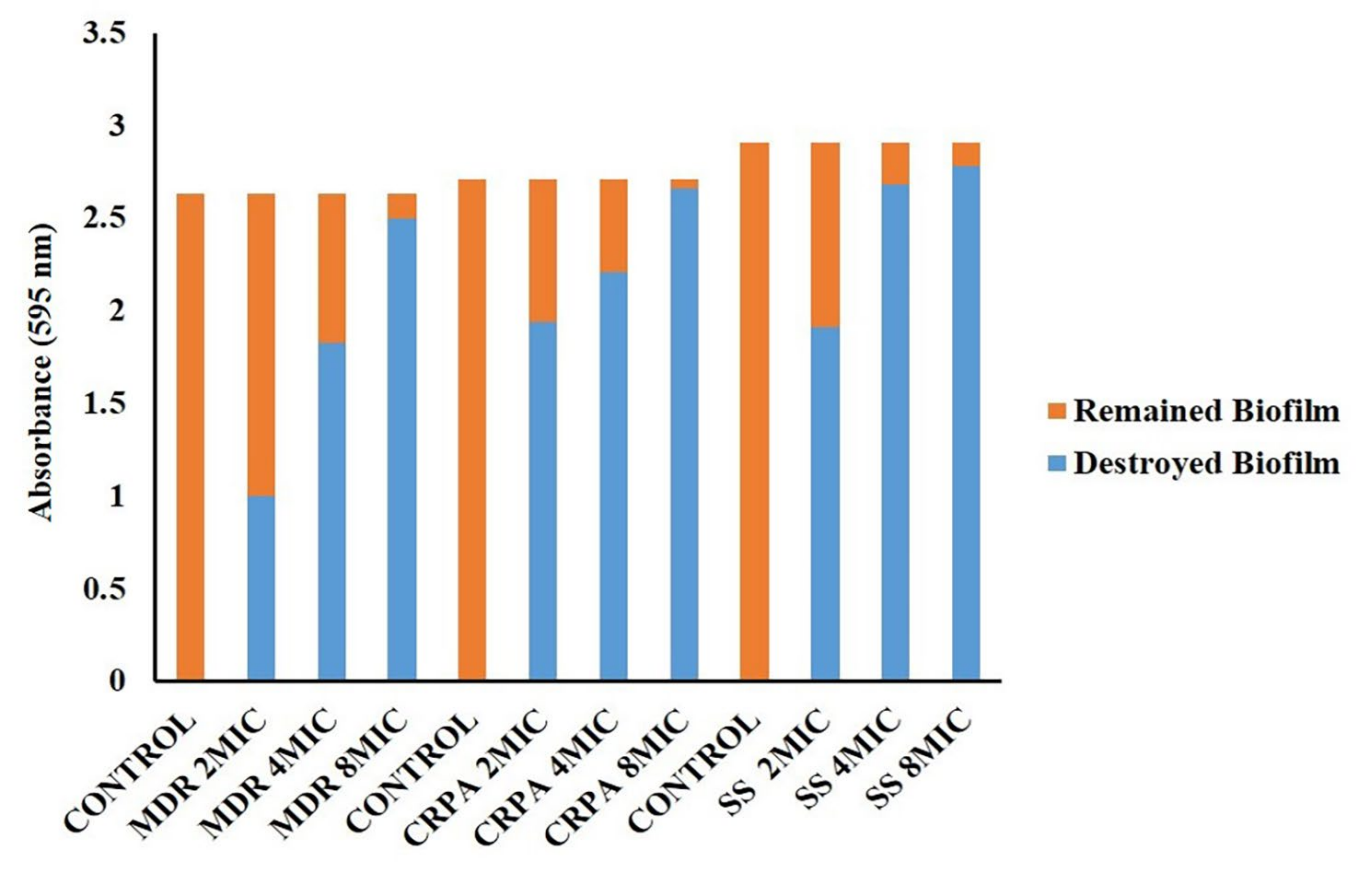
The effect of WLBU2 in different concentrations on dispersion of biofilm structure. MDR: Multidrug Resistant; CRPA: Carbapenem Resistant *P. aeruginosa;* SS: Standard Strain (ATCC 27,853)

There was a significant reduction in pre-existed biofilms of all *P. aeruginosa* isolates (MDR, CRPA, and ATCC 27,853) following treatment with 4× MIC of WLBU2. As shown in Figs. 3 and 32 µg/mL of WLBU2 was able to disperse 69.7% and 81.3% of MDR *P. aeruginosa* isolates and ATCC 27853*P. aeruginosa* biofilms, respectively (*P* < 0.03), while a 92.2% reduction in the CRPA biofilm was observed after treatment with 16 µg/mL WLBU2 (*P* < 0.03). WLBU2 at the concentrations of 2× MIC could not disperse structural integrity of MDR and standard (ATCC 27,853) *P. aeruginosa* biofilms (*P* > 0.05), whereas CRPA biofilms were dispersed at 2× MIC in CRPA (*P* < 0.05). Overall, we provide evidence to reveal that biofilms dispersal activity of 4× MIC of WLBU2 against preformed biofilm structure of MDR- and CRPA as well as ATCC 27,853 strains.

### WLBU2 alters the biofilm‑associated gene expression profiles of *P. aeruginosa*

**Fig. 4:**
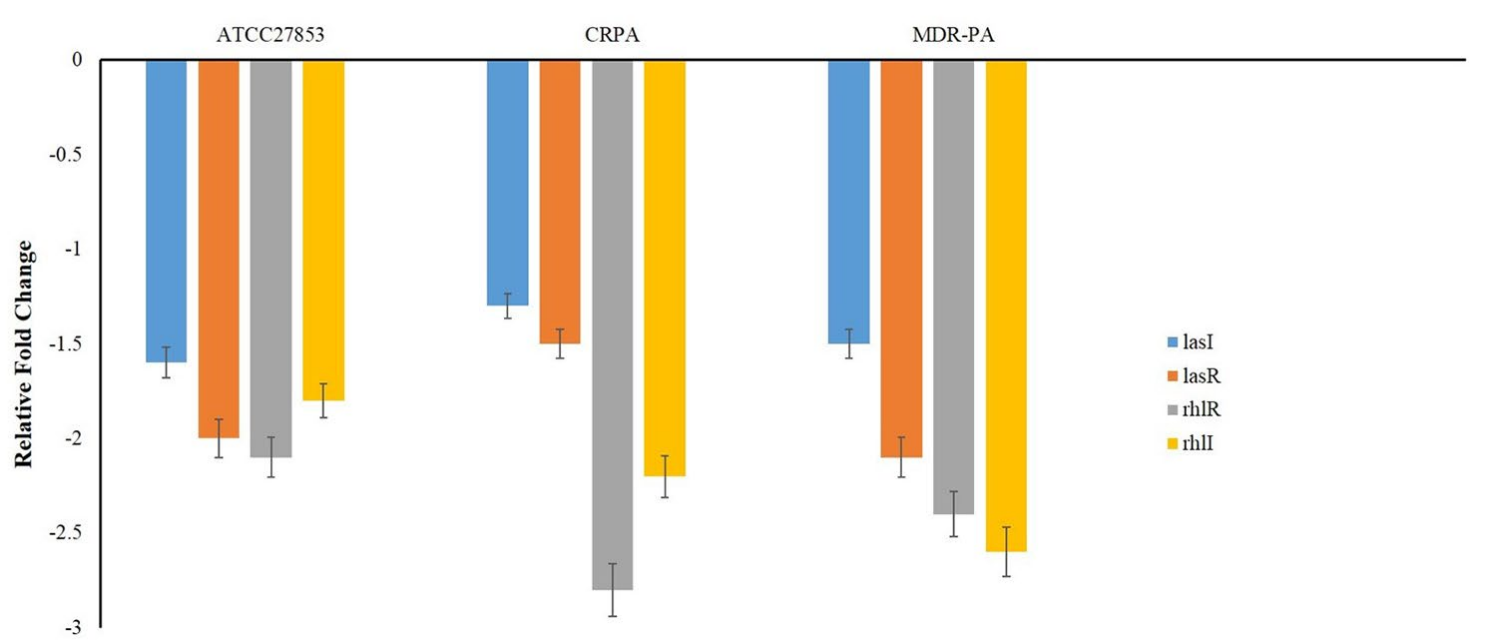
The expression levels of genes involved in *P. aeruginosa* biofilm formation influenced by WLBU2

To further test the effect of WLBU2 on biofilm formation, we examined the relative expression of biofilm-related genes (quorum sensing genes). The relative expression of biofilm-related genes was assessed using RT-qPCR in correlation to biofilm growth inhibition. According to the results in Fig. 4, the relative expression of *rhlI*, *rhlR*, *lasI* and *lasR* genes in isolates treated with 1/2 MIC of WLBU2 were reduced approximately 3 fold, compared to the untreated isolates (*P* < 0.05).

### Hemolytic activity

Results of Hemolytic activity assay showed that WLBU2 has no hemolytic activity against RBCs at concentrations of 5 to 200 µg/mL (*P* > 0.05). According to present data, WLBU2 was considered a safe and hemocompatible agent.

### Inhibitory effect of WLBU2 on biofilm formation in a murine catheter‑associated infection model

**Fig. 5:**
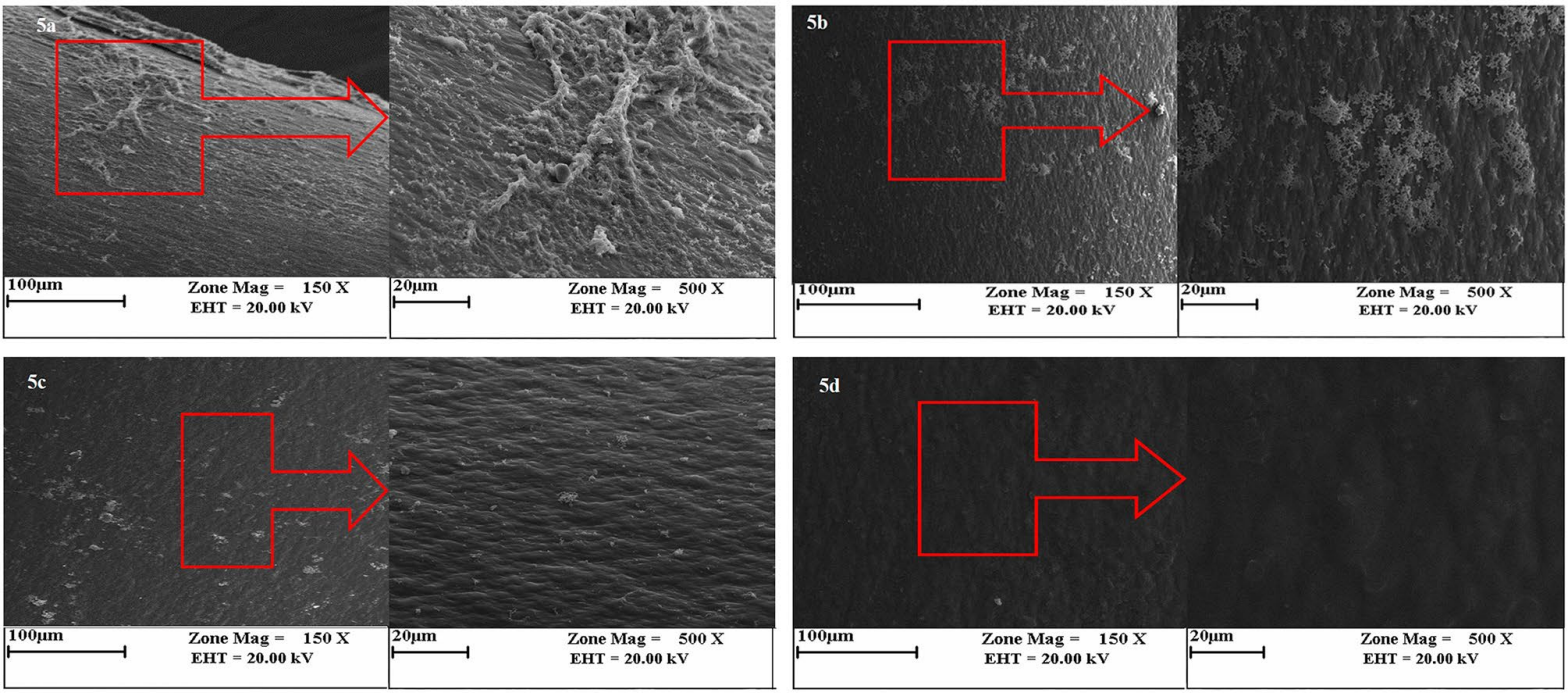
In vivo examination of CRPA biofilm biomass formed on the surface of catheters after treatment by different concentration of WLBU2; control (**5a**), treated biofilm at 1/4 MIC (**b**), 1/2MIC (**c**) and 1 MIC (**d**)

To investigate the *in-vivo* anti-biofilm effects of WLBU2, a murine catheter-associated CRPA infection model was used. Results showed that WLBU2 at 1/4×MIC, 1/2×MIC, and 1×MIC significantly decreased the biofilm formation in CRPA by 33%, 52%, and 67%, respectively (*P* < 0.05). To confirm the biofilm quantification by the colorimetric assay, SEM examination was used. Untreated biofilms (Fig. 5a) consisted of a denser network of exopolymeric matrix and microbial cells than treated biofilms at 1/4×MIC, 1/2×MIC, and 1×MIC of WLBU2 (Fig. 5b-d).

## Discussion

In the past years, the emergence and development of MDR bacterial infections have decreased therapeutic efficiency of conventional antibiotics. To overcome antibiotic resistance in bacteria, there is an urgent necessity for the discovery of novel antibacterial compounds with broad-spectrum activity and low cytotoxicity [22]. In this regard, one of the main strategies is the design and development of natural AMPs. The AMPs are potential antimicrobial agents and can overcome the limitations of conventional antibiotic agents [23]. WLBU2, a cationic synthetic peptide, has good activity against gram-positive and gram-negative bacteria [17]. In the present study, we aimed to study the antibiofilm effect of WLBU2 against MDR, CRPA, and standard (ATCC 27,853) strains of *P. aeruginos.*

Our findings revealed that MIC and MBC values of WLBU2 against MDR- and ATCC 27,853- *P. aeruginosa* strains were 8 and 16 µg/ml, respectively. On the other hand, our results revealed that both MIC and MBC against CRPA were 4 µg/ml. Furthermore, comparison of the killing kinetics of WLBU2 and colistin as a last-resort antibiotic against *P. aeruginosa* revealed rapid bactericidal activity of the peptide at 1*×* MIC. Following treatment with higher concentrations of WLBU2 and colistin, bactericidal activities were observed against *P. aeruginosa* in shorter time, which could be a promising property in combating severe infections. Therefore, WLBU2 could help in limitation of infections by potential pathogens in the first few hours following bacterial colonization. For an effective treatment, the incidence of resistance to WLBU2 should be low. Mostly the colistin resistance mutations were harbored by *P. aeruginosa* strains exhibiting pandrug resistance phenotype which can cause untreatable infections. Considering these facts there is an urgent need to develop novel therapeutic alternatives [24]. WLBU2 is a human cationic antimicrobial peptide with a broad antimicrobial activity [25].

Lin et al. used biofilm-inhibition assays to analyze bacterial adhesion and biofilm formation at sub-inhibitory concentration (1/3 x MIC) of WLBU2 by the crystal violet method. The expression level of biofilm-related genes was assessed using RT-qPCR for correlation with biofilm growth inhibition. They showed that conventional antibiotics at 1x MIC demonstrated modest ESKAPE biofilm prevention while 1/3 MIC of AMPs exhibited up to 90% biofilm prevention. Compared with colistin and LL37, WLBU2 was more effective in inhibiting bacterial adhesion. Changes in the expression level of biofilm-related genes were consistent with biofilm inhibition [17]. In a study by Chen et al. WLBU2 was compared with the human AMPs LL37 for (i) antibiofilm potency using *P. aeruginosa* on polarized human bronchial epithelial cells, and (ii) efficacy in murine *P. aeruginosa* pneumonia model using intratracheal delivery of bacteria and AMPs. They observed that WLBU2 (16 µM) inhibits biofilm formation by up to 3-log compared with 1-log decrease by LL37. With a single dose of 1 µg (0.05 mg/kg) instilled intratracheally, the initial effect of LL37 was moderate and transitory, as bacterial load and inflammatory cytokines enhanced at 24 h with observed signs of disease such as hypothermia and lethargy, consistent with moribund state requiring euthanasia. In sharp contrast, WLBU2 reduced bacterial burden (by 2 logs) and bacteria-induced inflammation (leucocytic infiltrates, cytokine and chemokine gene expression) at 6 and 24 h post-exposure, with no observed signs of disease or host toxicity [26].

Sweden et al. evaluated the activity of WLBU2 against MDR *Acinetobacter baumannii* and *Klebsiella pneumoniae* in planktonic cells and biofilm modes, alone and in combination with classical antimicrobial agents. In their study, to determine MBEC Calgary biofilm device was applied and to investigate the cytotoxicity of agents on eukaryotic cells the MTT assay was used. To evaluate the ability of WLBU2 to bind bacterial DNA, electrophoretic mobility shift assays were used.The MIC and MBC values of WLBU2 were same and ranged from 1.5625 to 12.5 µM. The Vero cells and human skin fibroblasts were observed to be viable at these concentrations. The MBEC of WLBU2 ranged from 25 to 200 µM. A significant reduction of eukaryotic cell viability was shown at the MBEC concentrations. Sub-inhibitory concentrations of WLBU2 in combination with amoxicillin-clavulanate or ciprofloxacin for *K. pneumoniae*, and with tobramycin or imipenem for *A. baumannii* exhibited a synergism effect that led to a significant reduction of MIC and MBEC. However, all combinations caused considerable decrease in viability of eukaryotic cells [27].

In the present study, we analyzed the hemolytic activities of WLBU2 peptide. The peptide at MIC level exhibited no hemolytic activity. These results suggest that the WLBU2 could be developed as a safe therapeutic agent. Furthermore, we revealed that at concentrations of 64–128 mg/ml WLBU2 can inhibit biofilm formation in strains with ability to develop strong biofilm, and can eradicate biofilm bacteria at 256–512 mg/ml. However, results indicated that to completely remove the bacteria enclosed in the biofilm, a concentration of several times higher than the MIC value of the peptide is required. We provide evidence to reveal biofilm dispersal activity of 4× MIC of WLBU2 against preformed biofilm structure of MDR *P. aeruginosa* -and CRPA as well as ATCC 27,853 strains. In general, AMPs can inhibit initial attachment, biofilm maturation and increase biofilm dispersal [15].

The ability of WLBU2 peptide to inhibit biofilm formation is due to effect of the peptide on bacterial membranes. It is revealed the WLBU2 peptide increase the permeability of the bacterial membranes and disruption [26].

In another study conducted by Lashua et al., Using abiotic and biotic biofilm assays, and co-culturing *P. aeruginosa* with polarized human airway epithelial cells, the ability of WLBU2 to inhibite biofilm formation alone and in combination with conventional antibiotics was investigated. They showed a dose-dependent decrease in biofilm development on an abiotic surface and in association with CF airway epithelial cells. WLBU2 inhibited biofilm formation when CF clinical isolates of *P. aeruginosa* co-cultured with mucus-producing primary human CF airway epithelial cells, even at low pH and high salt conditions that mimic the CF airway. When used in combination, WLBU2 significantly increases killing by conventional antibiotics tobramycin, ciprofloxacin, ceftazidime and meropenem [14].

It is presumed that AMPs down-regulate the expression of genes responsible for the transport of binding proteins and biofilm formation. Therefore, these peptides can suppress biofilm formation [21]. The results obtained from the semi-quantitative RT-PCR assay revealed that the expression levels of *rhlI*, *rhlR*, *lasI* and *lasR* genes in isolates treated with 1/2 MIC of WLBU2 were down-regulated by more than four-fold compared to the untreated isolates. Quorum sensing (QS) in *P. aeruginosa* is responsible to regulation of various virulence factors. Two complete QS systems including *las* and *rhl* have been recognized in *P. aeruginosa*. The *rhl* system consists of the transcriptional activator *RhlR* and the *RhlI*. Similarly, the *Las* system consists of *LasR* (transcriptional activator) and *LasI* (Autoinducer synthase enzyme).These two systems have a main role in biofilm formation and biofilm development [28, 29]. Studies have revealed that *lasI* and *rhl* mutant *P. aeruginosa* strains formed very thin and easily eradicated biofilms. These results stated that *las* and *rhl* QS systems are essential for biofilm development [30, 31].

Several studies have surveyed the effects of natural peptides on expression levels of biofilm-associated genes. For example, in a study performed by Rohde et al., it is revealed that expression of the *icaA*, *icaD*, and *icaR* genes which are responsible for *Staphylococcal* biofilms was down regulated by β-defensin 3 [32]. YU et al. have used RT qPCR to determine the effect of MC1 peptide on the expression levels of the genes encoding biofilm components in MDR *P. aeruginosa*. They showed that the relative expression of the genes *PslA*, *PelA* and *AlgD* was down regulated [33]. Shang et al. revealed that Trp-containing peptides inhibit biofilm formation by down regulating *pelA*, *algD*, and *pslA* genes in *P. aeruginosa* [34]. A study performed by Farshadzadeh et al. surveyed the antimicrobial and anti‑biofilm effects of dermcidin‑derived peptide DCD-1 L against *A. baumannii*. They showed that dermcidin‑derived peptide DCD-1 L down-regulates the biofilm-related genes and inhibit biofilm formation [21]. The finding of the present study revealed that in murine catheter-associated infection model, WLBU2 at the sub-lethal concentrations significantly inhibits the biofilm formation.

In conclusion, results revealed that WLBU2 peptide has a strong inhibitory and eradicative activity against the *P. aeruginosa* biofilm. Moreover. Our finding showed that to complete remove the bacteria enclosed in the biofilm, a concentration several times higher than the MIC value of the peptide is required. In addition, WLBU2 peptide down regulated the expression levels of genes involved in development and maturation of *P. aeruginosa* biofilm. The peptide did not exhibit any hemolytic activity against RBCs. Therefore, this peptide can inhibit biofilm formation without cytotoxicity and hemolytic activity. Considering the available data, WLBU2 can be considered as a safe and hemocompatible natural antibiofilm agent.

## Data Availability

All data was presented in this manuscript.

## Abbreviations

*AMP*: Antimicrobial Peptides
CRPA: Carbapenem Resistant *Pseudomonas aeruginosa*
*MIC*: Minimum Inhibitory Concentration
*MBC*: Minimum Bactericidal Concentration
QS: Quorum Sensing
MBEC: Minimum Biofilm Eradication Concentration
SEM: Scanning Electron Microscope
